# The Effects of Exergaming Training on Balance in Healthy Elderly Women—A Pilot Study

**DOI:** 10.3390/ijerph18041412

**Published:** 2021-02-03

**Authors:** Anna Brachman, Wojciech Marszałek, Anna Kamieniarz, Justyna Michalska, Michał Pawłowski, Anna Akbaş, Grzegorz Juras

**Affiliations:** Institute of Sport Sciences, The Jerzy Kukuczka Academy of Physical Education in Katowice, 40-065 Katowice, Poland; w.marszalek@awf.katowice.pl (W.M.); a.kamieniarz@awf.katowice.pl (A.K.); j.michalska@awf.katowice.pl (J.M.); m.pawlowski@awf.katowice.pl (M.P.); a.akbas@awf.katowice.pl (A.A.); g.juras@awf.katowice.pl (G.J.)

**Keywords:** virtual reality, elderly, balance, exergaming, exercise training

## Abstract

Our aim was to observe, through objective testing using an assessment module incorporated in a new exergaming system, whether elderly people’s static and functional balance is improved by a balance exergaming training program based on movements performed in everyday life. Thirteen healthy elderly women participated in 12 sessions of balance-based exergaming training (three times a week, 30 min per session). All objective outcomes, the quiet standing test, functional balance test (FBT), and limit of stability (LOS) test, were measured on three occasions: before intervention, after six training sessions, and after the completion of the four-week program. The results showed a significant improvement in LOS performance after the intervention. In FBT, participants exhibited a significant decrease (*p* < 0.01; Kendall’s W = 0.5) in the average time to target hit after six trainings. The average center of pressure velocity increased after six and 12 sessions, however did not reach significance (*p* = 0.053); nevertheless the size of the effect was large (η_p_2 = 0.22). The parameters of the quiet standing test were not significantly affected by the training. The results support the need for more definite and objective studies assessing exergaming for balance in elderly.

## 1. Introduction

Postural control is the basis for the execution of movements in everyday life and is influenced by the visual, vestibular, and the somatosensory systems [1]. Reduced balance control, on the one hand, is a natural consequence of ageing; however, on the other hand, it substantially affects functioning and quality of life in elderly people. Furthermore, it is strongly associated with an increased risk of falls [2,3,4,5].

Recently, the gaming industry has developed various accessible commercial exergaming systems (VR). Augmented feedback about performance allows for individualized repetitive training of motor tasks in virtual reality environment, which, in turn, allows for exercise with higher intensity [6,7]. Motor-cognitive dual tasking in exergames provides additional challenges and supports motor learning processes [8]. Therefore, it is not surprising that in recent years the use of VR has been introduced in the field of rehabilitation in the elderly population [8,9].

A growing number of studies have demonstrated the positive effects of VR training on the elderly with regard to gait, balance, and cognitive function [10,11,12,13,14,15]. However, very few studies provided objective and quantitative evaluation. Balance tests generally used in clinical practice provide mostly qualitative, positive (disorder), or negative (no aberration from often arbitrarily accepted norm) results. The tests themselves are subjective and imprecise, and do not allow for assessment of the degree of disorder’s progression or improvement after an implemented treatment. They also often do not allow for comparison of the results in various groups of patients [16]. In a very recent review and meta-analysis [17], authors emphasized the need to develop study designs that utilize biomechanical and other objective measures of balance. Only four out of 16 included papers involved objective evaluation. In previous meta-analysis [18] on six included papers, none of them were using quantitative measures of postural stability. Objective assessment of motor performance after VR therapy is especially relevant for the possibility of quantifying improvement and needs accurate and objective instruments [19]. The search for and development of quantitative and objective measures of balance is also in line with evidence based medicine policy [20].

Although previous studies contributed to our understanding of the effects of implementing the VR as a rehabilitation tool in healthy elderly adults, further research is needed to examine the effects of implementing balance-based exergaming training on quantitative biomechanical measures of balance.

Considering all the above, we decided to create a novel system that would allow clinicians to objectively measure the postural components of balance with known and established measures of static and dynamic balance. Therefore, in the current experiment, we integrated two devices: a posturographic platform and a 3D measurement system. It also allowed us to embed a wider spectrum of movements into the created exergames, which could be controlled during exercising. The goal of this pilot study was to observe through objective testing whether healthy elderly people’s static and dynamic balance improved after a four-week balance-based exergaming training program.

We therefore hypothesized that exergaming training would induce significant improvements in the selected objective measures of balance even though the participants had been chosen from the healthy older adults population.

## 2. Materials and Methods

### 2.1. Study Design

The study was carried out as a quantitative trial with an intervention period of four weeks (pretest–training–posttest design).

### 2.2. Participants

The study involved a group of 13 healthy elderly women, members of the Silesian University of the third age. All participants met the following inclusion criteria: (1) age 65 years and older, (2) the ability to perform the measurement and training without any aids, and (3) no experience in virtual reality gaming. The exclusion criteria were as follows: (1) chronic systemic and inflammatory diseases (i.e., rheumatoid arthritis or osteoarthritis); (2) neurological, cardiovascular, or orthopedic diseases affecting postural stability (i.e., severe low back pain, epilepsy, uncontrolled hypertension, vestibular disorders); (3) diagnosed dementia; or (4) vision deficits.

Twenty three participants were screened prior to enrollment, five participants did not meet inclusion and exclusion criteria, three participants withdrew (due to schedule conflict), and another two participants declined to sign the consent form.

The study was carried out in accordance with the guidelines proposed in the Declarations of Helsinki and it was approved by the Institutional Ethics Committee (no. KB/28/2014). It was conducted from March 2018 to June 2018 at the Human Motor Behavior laboratory of the Academy of Physical Education in Katowice. All enrolled participants provided an informed consent. The participants were also informed that they could leave the study at any time without any explanation. The results of previous studies [21,22,23,24,25] indicate that posturographic variables in static and dynamic tests in healthy elderly people remain stable over time (6, 9, 12, or 14 weeks). Hence, based on results previously published in the literature, we decided not to include the control group in our preliminary research.

### 2.3. Intervention

In accordance with the results obtained in recent meta-analysis concerning VR balance training in the elderly [18], the participants in the current experiment received a total of 12 training sessions (three trainings per week), each training lasted for 30 min. All objective outcomes (postural stability and functional balance tests) were measured on three occasions: before intervention (baseline values), after six training sessions, and after the completion of the four-week program.

**Figure 1:**
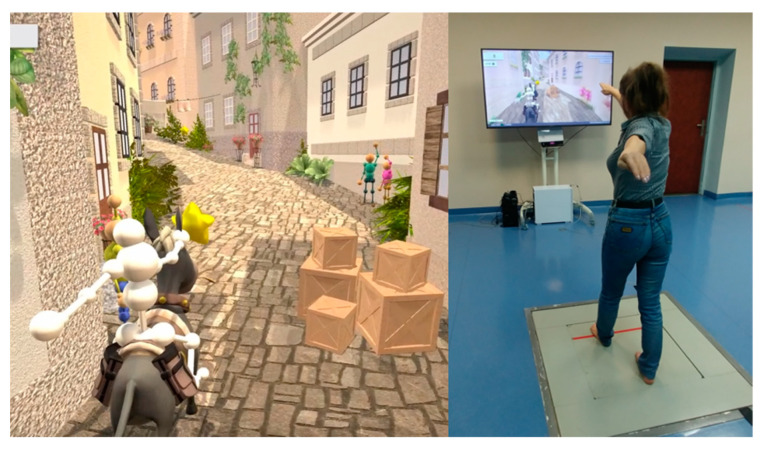
Virtual reality exergaming system setup.

**Figure 2:**
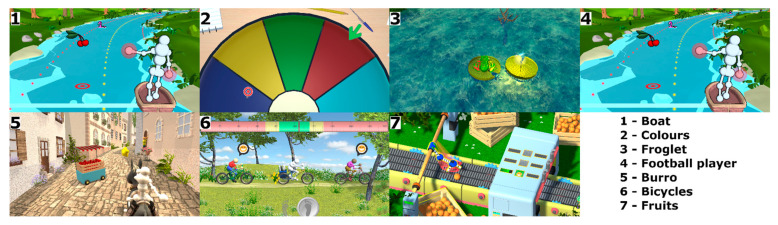
Games implemented in the virtual reality environment.

The VR balance training system included two integrated devices: a custom-made force platform and a 3D measurement system based on time-of-flight cameras (Kinect sensor system). Both devices were used to control and verify movement execution (Figure 1). Seven games were created for training balance (Figure 2), designed to facilitate and train crucial movements for elderly people.

Through avatar technology, images were projected on a 65-inch screen, which was situated 2 m away in front of the participant. The participants were introduced into the game as an avatar character, which provided instantaneous visual feedback about the patient’s movement execution. In each training session, the participants practiced static posture, dynamic weight shifting, single leg standing, leaning in different directions, trunk rotation, and taking steps. The games were adapted to the subject’s individual capabilities.

To avoid any bias, for the duration of our study, we asked participants not to be engaged parallel in any other organized physical training exercise program. The time of day that examination and trainings were taken was adjusted for each participant and was constant throughout the experiment. The physiotherapist supervised for safety during each session and participants were asked to report any indisposition or side effect.

### 2.4. Outcomes

In order to evaluate the effectiveness of the training program, a special diagnostic module was created. We decided to choose two well-known static and dynamic posturographic tests (limit of stability and quiet standing test) for assessing balance and create one more complex task. Thus, the module comprised three objective tests: quiet standing (QS), limit of stability (LOS) in the forward direction, and functional balance test (FBT). The LOS test in the forward direction and FBT were used to document dynamic balance performance, whereas the quiet standing testing was used to assess static balance. All measurements were done on a force platform (VB-Clinic VBC PP 0001), which was part of the VR system and by which the vertical ground reaction forces were registered at a 100 Hz sampling frequency.

The raw data from the platform was processed offline using the Matlab r2017b software (Mathworks Inc., Natick, MA, USA) with a low-pass, fourth-order Butterworth filter, and with a cut-off frequency of 7 Hz. Each participant completed a practice session for each test prior to balance measurements.

The LOS test is a widely used measure in biomechanical analysis of dynamic balance. Furthermore, it shows a significant positive correlation between the SOT composite score and anterior displacement on the LOS test for fallers [26,27].

The LOS test was repeated three times and divided into three phases: first phase—quiet standing, second phase—transition to maximum leaning position, third phase—maintenance of the maximal forward-lean [28]. In this task, the participants were asked to stand quietly during the first 10 s of the trial; then, after the signal tone, to lean in the forward direction as quickly as possible without moving the feet and to maintain this position until the end of the trial.

**Figure 3:**
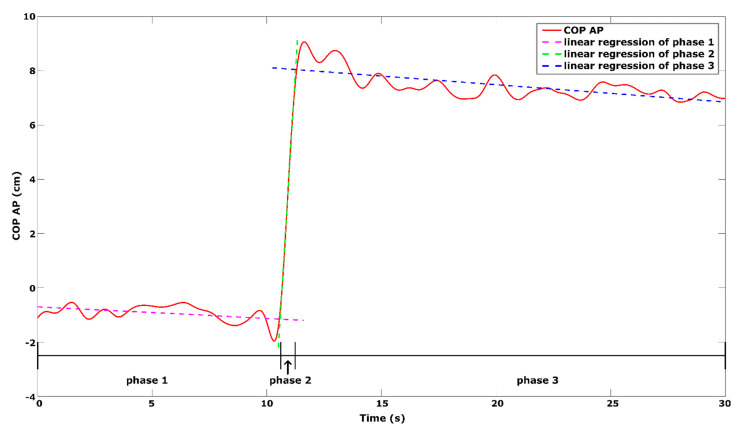
Phases representing subject’s COP excursion with regression lines during a limit of stability test.

Previous study has revealed a very high level of internal consistency of LOS test in group of healthy older adults (intra- and intersession ICC ranged between 0.82 to 0.99) [29]. In order to exclude the influence of the starting position (i.e., leaning forward during the quiet standing phase) on the range of the forward lean, the COP position was normalized to the participants’ medial malleolus position on the force platform (for details see [30]). The following variables were analyzed: mean COP position relative to the medial malleolus in the first (meanCOP1) and third phase (meanCOP3—range of forward lean) and leaning rate (LR—defined as linear regression coefficient of the 2nd phase) (Figure 3).

**Figure 4:**
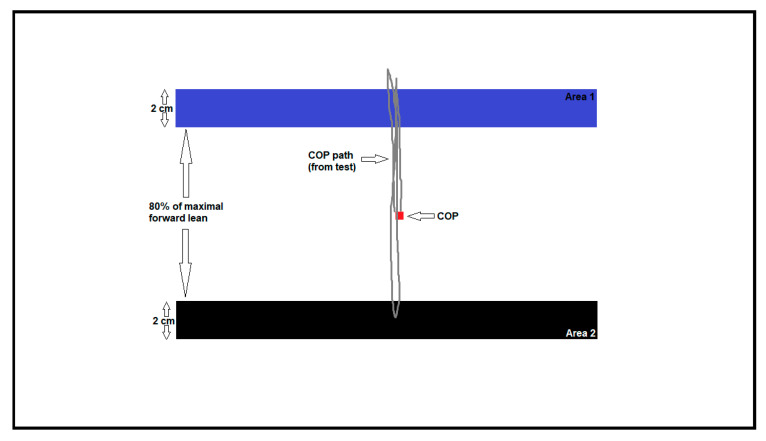
The functional balance test. Participant has to rapidly shift the center of foot pressure (COP) to reach the black area. Grey line shows the COP displacement during the measurement.

In the functional balance test (FBT), participants had to rapidly shift the COP to areas indicated on the screen by leaning the body without moving the feet (Figure 4).

The COP point was drawn in real time on the screen of the monitor placed in front of the participant. During the test, two areas with specific width and distance between them were alternately appearing. The experiment performed with healthy participants served to determine which width and distance would give the best reliability. The highest reliability was obtained for conditions where the width was 20 mm and the distance between areas was equal to 80% of the participant’s maximum forward lean (ICC ≥ 0.95) [31]. The FBT trial was repeated three times, and the following parameters were analyzed: average COP velocity (FvCOP), average time of target hit (FtCOP), and movement optimization (FoCOP). Movement optimization is defined as the amount of movement in the intended direction plus the amount of extraneous movement. A FoCOP score of 0% indicates that the participant did not deviate from a straight path during the test.

During quiet standing testing, the participants were asked to stand barefoot as still as possible on the force platform, maintaining their arms by their side while keeping their head straight. A total of six trials were performed: three trials with eyes open with the gaze fixated at a reference point located 2 m away in front of the platform and three trials with eyes closed. The quiet standing trials lasted for 30 s. The following main postural sway measurements were analyzed: sway ranges (raCOP) and center of pressure velocity (vCOP) with its directional subcomponents—anteroposterior (ap) and mediolateral (ml). The validity and reliability of the quiet standing test among healthy older adults have been previously established [29,32,33,34].

### 2.5. Statistical Analysis

The time course of average changes in dependent variables during the three sessions were analyzed using a one-factorial repeated measures analysis of variance (ANOVA) with Tukey (honestly significant difference) post hoc comparisons or a Friedman ANOVA with Bonferroni–Dunn post hoc test according to the normal distribution, if necessary. Levene’s test was used to assess the assumption of variance homogeneity. The assumption of sphericity was assessed using Mauchly’s test. Statistica software package version 13.1 for Windows (StatSoft, TIBCO Software Inc., Palo Alto, CA, USA) was used for all statistical procedures. An a-level of *p* < 0.05 was accepted as statistically significant.

Effect sizes were calculated to infer the importance of differences: small (0.01), average (0.06), and large (0.14) [35]. To improve the comparability of effect sizes between studies, less biased, partial eta squared (η_p_2) effect size was computed [36]. For non-parametric tests, Kendall’s coefficient was calculated.

### 2.6. Sample Size

No sample calculation has been made. However, as this is a pilot study, and to the author’s knowledge no previous studies using the two different systems (force platform and Kinect) combined together has been carried out, a sample size calculation based on previous work could not be done.

## 3. Results

Thirteen healthy elderly women were enrolled in the study. None of the participants dropped out from the study, and all of them received full allocated training. The mean age was 70.2 (range 65–80) years, mean height was 1.63 m (range 1.54–1.68 m), mean weight was 73.9 kg (range 57–90 kg), and mean body mass index was 27.76 kg/m^2^ (range 21.2–34.0 kg/m^2^). None of the participants reported any adverse events during the intervention.

**Figure 5:**
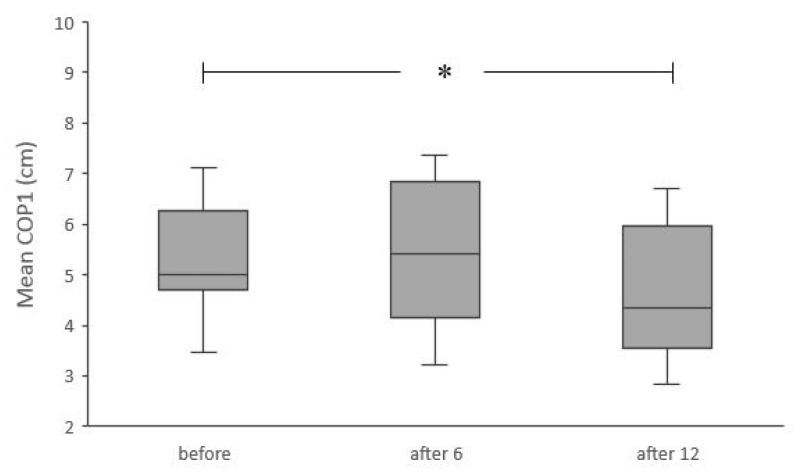
Results for the mean COP position relative to the medial malleolus in the first phase of limit of stability test, before intervention and after six and 12 training sessions. * Significant differences (*p* < 0.05). Box plots indicate interquartile ranges (areas within a box), medians (horizontal line in the box), error bars indicate the range (min–max).

**Figure 6:**
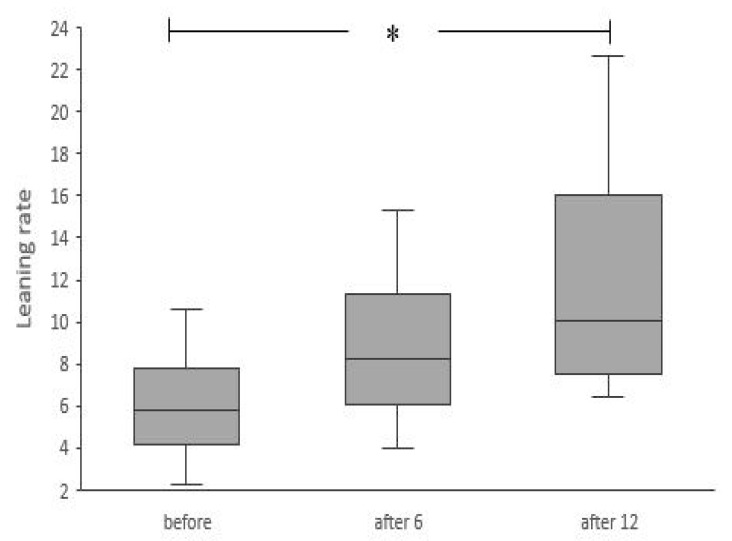
Results for the leaning rate (linear regression coefficient of the second phase of limit of stability test), which defines the speed of forward leaning movement, before intervention, after six and 12 training sessions. * Significant differences (*p* < 0.05). Box plots indicate interquartile ranges (areas within a box), medians (horizontal line in the box), and error bars indicate the range (min–max).

The results showed a significant improvement in the LOS test after 12 training sessions. Significantly lower values of mean COP1 (χ^2^ANOVA = 6.61, *p* < 0.05, Kendall’s W = 0.25) and significantly higher values of leaning rate (χ^2^ANOVA = 8.7, *p* < 0.05, Kendall’s W = 0.34) were registered after 12 training sessions (Figure 5 and Figure 6). The range of forward lean was not significantly affected by the training (F(2, 24) = 0.5, *p* > 0.05).

The results of FBT are presented in Table 1. The participants exhibited a significant decrease in FtCOP (χ2ANOVA = 12.92, *p* < 0.01, Kendall’s W = 0.497) after six trainings when compared to the baseline values. After 12 sessions it increased slightly, so that the difference did not remain significant; nevertheless, the size of the effect was large. Average COP velocity increased after six and 12 training sessions; however, this change did not reach significance (F(2, 24) = 3.32, *p* = 0.053, η_p_2 = 0.22), while the size of the effect was also large. Movement optimization was not significantly affected by the training (F(2, 24) = 1.34, *p* > 0.05).

The results of the quiet standing test are shown in Table 1. No significant difference between the baseline and six and 12 training sessions was found.

## 4. Discussion

The goal of this pilot study was to observe through objective testing whether healthy elderly people’s static and functional balance improved after a four-week balance-based exergaming training program. The main finding emerged from this study: a relatively short, balance-based exergaming training readily available for elderly people can improve certain aspects of their balance, even though they belong to healthy population. To the authors’ knowledge, this is the first time when an exergaming system used to train balance combines two different gaming and measurement equipment (force platform and Kinect) and, at the same time, includes both training and objective assessment modules. This pilot study evaluated the effect of a four-week exergaming training program on balance in untrained, healthy, elderly women with using quantitative measures of balance. As highlighted by previous authors [11,37,38], force-plate posturography remains the safest and most appealing method of evaluating balance. It has major advantages such as simplicity, objectivity, more precise determination of changes in balance parameters, and more accurate reliability [8].

The results revealed improvements in measures of dynamic balance during the exergaming intervention training period. The linear regression coefficient in the second phase of the LOS test is determined by the participants’ velocity of movement [28]; thus, increased LR values after 12 trainings indicate that the participants could transfer their center of mass more dynamically. Sapi et al. [15] similarly observed significant increase in velocity of LOS performance in conventional and Kinect training groups when compared to the control group (no intervention); however, when compared to baseline values, the movement velocity was significantly higher after the intervention only in the Kinect group. This is in line with the results obtained by previous authors [11]. As leaning movement velocity decreases with age, perhaps also due to the slowing of reflexes [4], this change suggests a significant improvement in the participants’ mobility and movement control.

The values of the mean position of COP in the first phase of the LOS test decreased significantly. These results indicate that after intervention, the participants’ COP was closer to the malleolus position. This, in turn, means that after intervention the participants were standing in a more upright position. To the authors’ knowledge, this is the first time where the COP position was normalized in the LOS test; thus, it is not possible to compare these results with other authors. However, previous findings revealed that both PD patients and elderly people move their COP during standing towards the anterior boundary of stability [37]. Such a strategy results in a flexed posture, which is intended to provide protection against backward falls [39]. Thus, the transition of the participants’ mean COP position further from the anterior boundary of stability possibly indicates that functional balance in this area improved. Contrary to the results obtained by previous authors [11], the lean range did not change significantly in current study. Unfortunately we cannot compare directly our results with these of Duque et al. [8] and infer whether previous authors obtained significant increase in forward direction, because authors did not report forward lean range but the area of limits of stability (cm^2^). We propose, however, that lack of significant change in range of forward lean in current study could result from the normalization of the COP position in relation to the medial malleolus. If the COP position were not normalized in the third phase of the LOS test, because of the different starting position, the forward lean range would be bigger after the intervention (as the results indicate that after 12 trainings, the participants in the first phase of the LOS test shifted their COP by 0.71 cm on average in a more backwards direction).

The outcome of FtCOP during the functional balance test depends on the change of at least one of three components: the participant’s reaction times, movement velocity, and length of sway path. We propose that in the current experiment, the first two components influenced the outcome of FtCOP. Although the average COP velocity did not reach significance, the size of the effect was large. We believe that this “nearly significant” (*p* = 0.053) outcome results from a small sample size. Furthermore, as the sway path (herein defined as FoCOP) did not change, the improvement of FtCOP could be the result of the participants’ decreased reaction times. This finding is in line with the work of Lajoie [40] and Bisson et al. [41] and supports their suggestions that completing a training program allows participants to improve their reaction time in a postural reaching task as less attention is needed for the standing task. The training program used in the current study involved various exercises that strongly challenge postural control and are included in whole-body movements. Additionally, constant augmented feedback about performance and graded complexity may have contributed to the positive effects of the training reflected in the dynamic balance performance.

Both postural sway measures in quiet standing test, COP velocity and range of sway, did not change significantly in any of the directions after the completion of the VR balance-based training. In the literature concerning postural stability, it is generally stated that during quiet standing, lower postural sway parameters (i.e., range or velocity) are interpreted as better stability [37,38,39]. There are varied reports regarding influence of VR training on postural control during quiet standing task in healthy elderly; however, most of the previous studies observed that it does not alter participants’ sway behavior [13,40,41,42,43]. In contrast, some researchers have shown reductions of average body sway in eyes closed condition after VR intervention and conventional training in ap and ml directions [44]. Park et al. [45] observed decrease in total sway length and average sway speed after VR and ball exercise training during standing with eyes open. Pluchino et al. [46], in turn, observed increase in COP area after implementing VR, tai chi, and balance-training in each group. These discrepancies may result from differences in the methodology of the conducted research. For example, Park et al. [45] conducted one 30 s trial with gazing forward on the top of an analysis system with biofeedback. Pluchino et al. [46] conducted three 10 s trials and Ordnung et al. [44] three 15 s trials. In most cases, authors did not report sampling frequency, which has a strong impact on several parameters, such as confidence ellipse area. Additionally, measurement time, which varied across studies, directly determines length of sway path.

It might be surprising that postural stability did not change after the implementation of the balance training; however, as highlighted by Esculier et al. [43], using a more complex task while standing on the force platform (i.e., a dynamic task or foam interface) would have been more sensitive to balance changes induced in healthy elderly people. Our results seem to confirm these findings.

This study has limitations that should be addressed. First, no sample calculation has been made. However, as this is a pilot study, and to the author’s knowledge no previous studies using the two different systems (force platform and Kinect) combined together has been carried out, a sample size calculation based on previous work could not be done. Another limitation is that this pilot study did not include a control group; thus, any improvements in the participants’ balance according to traditional balance training could not be evaluated. This pilot study shows very promising results. However, the small number of participants warrants further and more extensive research, which would include the involvement of both sexes and control groups with different type of exercises. Moreover, to set up a more precise treatment routine, further research on investigating the exact amount of intensity, time, and frequency of trainings would be necessary, since our aim is to provide long-lasting balance interventions. Our readily available system presents an opportunity to enhance a short term care programs for elderly people, which would attract and encourage them to take up physical activities. At the same time, it gives clinicians way of objective and valid balance assessment.

## 5. Conclusions

This study highlights the importance of implementing balance training for older adults. The results demonstrate that even a relatively short four-week training period can significantly improve functional balance in healthy elderly women. At the same time, this type of VR training does not influence static balance during quiet standing task. Measures used to assess balance in healthy participants after implementing virtual reality balance training should be strongly considered as well.

The results indicate that the LOS and FBT tests are sensitive enough to reveal even subtle changes in participants balance induced by VR training in healthy elderly women.

## Figures and Tables

**Table 1:** The results of the functional balance test and quiet standing test.

Variables	Baseline	After 6 Session	After 12 Session	*p*-Value	Effect Size
	$\bar{x}\;\pm \;\mathrm{SD}\;$ Me (IQR)	$\bar{x}\;\pm \;\mathrm{SD}\;$ Me (IQR)	$\bar{x}\;\pm \;\mathrm{SD}\;$ Me (IQR)		
Functional Balance Test					
FtCOP (s)	2.69 ± 0.5 2.52 (2–3)	2.28 ± 0.3 * 2.27 (2–3)	2.38 ± 0.3 2.34 (2–3)	0.001	0.49
FvCOP (mm/s)	64.86 ± 16.7 59.66 (53–73)	73.87 ± 17.1 69.45 (63–82)	73.87 ± 15.4 74.56 (66–81)	0.053	0.22
FoCOP (%)	216.24 ± 67.2 205.46 (166–267)	188.65 ± 70.5 204.34 (174–212)	213.03 ± 59.8 199.78 (177–242)	0.28	0.1
Quiet Standing Eyes Open					
raCOP_ap (mm)	22.81 ± 7.34 23.11 (21–26)	23.83 ± 9.36 26.04 (15–28)	24.29 ± 6.39 24.95 (18–29)	0.64	0.03
raCOP_ml (mm)	11.9 ± 4.32 12.81 (8–16)	10.31 ± 4.03 9.05 (8–14)	12.8 ± 6.31 11.04 (10–13)	0.10	0.07
vCOP_ap (mm/s)	8.22 ± 3.4 8.02 (6–10)	8.15 ± 3.12 6.83 (6–11)	7.9 ± 2.74 7.5 (6–11)	0.77	0.02
vCOP_ml (mm/s)	4.66 ± 2.27 4.36 (3–7)	3.83 ± 1.49 3.27 (2–5)	4.55 ± 2.95 4 (3–5)	0.36	0.07
Quiet Standing Eyes Closed					
raCOP_ap (mm)	24.43 ± 9.43 21.33 (21–28)	24.62 ± 8.45 22.97 (18–33)	24.44 ± 6.76 23.17 (22–26)	0.99	0.005
raCOP_ml (mm)	12.79 ± 4.85 12.52 (9–16)	11.89 ± 5.12 10.21 (9–13)	13.09 ± 8.01 10.33 (8–15)	0.92	0.02
vCOP_ap (mm/s)	11.88 ± 6.47 10.22 (8–13)	11.56 ± 6.1 8.86 (8–13)	10.73 ± 5.41 9.4 (7–12)	0.11	0.07
vCOP_ml (mm/s)	5.52 ± 2.8 4.68 (3–8)	4.58 ± 2.23 4.2 (3–6)	5.14 ± 3.46 4.65 (3–6)	0.19	0.09

SD = standard deviation; ME = median; IQR = interquartile range; FtCOP= average time of target hit; FvCOP = average COP velocity; FoCOP= movement optimization; raCOP = sway range; vCOP = sway velocity; ap = anteroposterior; ml= mediolateral; *p*-value for repeated-measures ANOVA or Friedman test * Differences statistically significant between Baseline vs After 6 Session (*p* < 0.05). Effect size: partial eta squared (η_p_2) or Kendall’s coefficient (W), small (0.01), average (0.06), and large (>0.14).

## Data Availability

The data can be accessed through the Open ICPSR data repository service at https://doi.org/10.3886/E117863V1.
